# Supplementary material for: Engineering the Aggregation of Dyes on Ligand-Shell Protected Gold Nanoparticles to Promote Plexcitons Formation
Source: Nanomaterials (Basel). 2022 Apr 1;12(7):1180. doi: 10.3390/nano12071180 (PMC9000468; doi:10.3390/nano12071180)
Supplement: Supplementary file 1 [file nanomaterials-12-01180-s001.zip › nanomaterials-1644000-supplementary.pdf]

nanomaterials

Supplementary Materials

Engineering the Aggregation of Dyes on Ligand-Shell Protected Gold Nanoparticles to Promote Plexcitons Formation

Nicola Peruffo [1], Giovanni Parolin [1], Elisabetta Collini [1,2,*], Stefano Corni [1,*] and Fabrizio Mancin [1,*]

[1] Department of Chemical Sciences, University of Padova, via Marzolo 1, 35131 Padova, Italy; nicola.peruffo@unipd.it (N.P.); giovanni.parolin@studenti.unipd.it (G.P.)
[2] Padua Quantum Technologies Research Center, via Gradenigo 6, 35131 Padova, Italy
* Correspondence: elisabetta.collini@unipd.it (E.C.); stefano.corni@unipd.it (S.C.); fabrizio.mancin@unipd.it (F.M.)

Table of Contents

S1. Experimental Procedures……………………………………………………………...S1
S2. Synthesis and Characterization of Thiol Monolayer Protected Small Gold Nanoparticles (SNPs)…………………………………………………………………………..S2
S3. Table of Dynamic Light Scattering Analyses of NPs and Their Hybrids…………...S3
S4. Further Computational Procedures…………………………………………………..S4
S5. Calculation of the H_2TPPS^{2-} Configuration on BNPs Surface……......………………S14
References……………...……………………………………………………………….S15

S1. Experimental Procedures

General: Solvents were purified by standard methods. All commercially available reagents and substrates were used as received. The thiol used as a capping agent was synthesized with a procedure described in the literature [1]. The synthesis and characterization of BNPs were reported elsewhere [2]. All the solvents and reactants were obtained from Sigma-Aldrich (Merck KGaA, Darmstadt, Germany) and used as received without further purification unless stated otherwise. The 4,4′,4″,4‴-(Porphine-5,10,15,20-tetrayl) tetrakis(benzenesulfonic acid) was bought by PorphyChem.

TEM analysis was performed with a Jeol 300 PX electron microscope (JEOL, Tokyo, Japan) and the collected images were analysed with ImageJ software. Thermogravimetric analysis (TGA) was run on 1 mg nanoparticle samples using a Q5000 IR model TA (TA instruments, New Castle, DE, USA). The measurement was set to reach 100 °C and to keep them for 10′, in order to remove completely the water traces, and then to 1000 °C under a continuous air flow.

NMR spectra were recorded using a Bruker AV III 500 spectrometer (Bruker, Billerica, MA, USA) operating at 500 MHz for ^{1}H. Chemical shifts are reported relative to internal Me_4Si.

A Zetasizer Nano ZS (Malvern Panalytical Ltd, Malvern, UK) was used for DLS measurements. The instrument was equipped with a 633 nm laser and a detector in configuration NIBS (non-invasive backscatter system—173°). The temperature was kept at 25 °C during the measurement and 0.197 was used as the refractive index of the NPs. Zetasizer software was used to analyse the data.

UV-Vis extinction spectra were performed with a Varian Cary 100 scan spectrophotometer (Agilent Technologies Inc., CA, USA) in a Quartz cuvette with a reduced optical path length of 1 cm × 0.2 cm (Madatech®, Milano, Italy). The optical path for the absorbance measurements was 1 cm.

The luminescence spectra were recorded with a fluorimeter Fluoromax-P (Horiba Ltd., Kyoto, Japan). The measurements were performed in a Quartz cuvette with a reduced optical path length of 1 cm × 0.2 cm (Madatech®, Milano, Italy). All the spectra

were recorded by setting at 10 nm and 2.5 nm the entrance and exit slits, respectively; each data point has an integration rate of 0.1 s if not stated otherwise.

S2. Synthesis and Characterization of Thiol Monolayer Protected Small Gold Nanoparticles (SNPs)

Small gold nanoparticles were prepared according to a previously reported two-step procedure [3]. Glassware in contact with gold nanoparticles was washed with aqua regia before and after its use and rinsed with distilled water.

A solution of $HAuCl_4{\cdot}3H_2O$ (50 mg, 0.127 mmol, 1 equiv) in water (2 mL) was extracted with a solution of tetraoctylammonium bromide (0.175 g, 0.318 mmol, 2.5 equiv) in N_2 purged toluene (125 mL). Dioctylamine (0.613 g, 2.539 mmol, 20 equiv) was added to the resulting reddish-orange organic solution (the amount of dioctylamine was calculated in order to obtain 2 nm nanoparticles). The mixture was vigorously stirred under N_2 for 3.5 h. During this period, the colour of the mixture faded. Then the solution was cooled at 0 °C and a $NaBH_4$ solution (47.0 mg, 1.257 mmol, 10 equiv) in H_2O (0.5 mL) was then rapidly added. The color of the solution turned rapidly to black and after 1.5 h of stirring at 0 °C, the aqueous layer was removed. To the obtained nanoparticle solution, the thiol (0.123 mmol, 1 equiv) dissolved in 2 mL of MeOH was rapidly added. The reaction mixture was stirred overnight at 0 °C. All the formed AuNPs were insoluble in toluene, hence the solvent of the reaction was poured out and the sample was rinsed 3 times with toluene. Successively, it was centrifuged four times, using MeOH and toluene. The resulting NPs were finally purified by gel permeation chromatography with Sephadex G-25 (Merk KGaA, Darmstadt, Germany).

S2.1. Characterization of Nanoparticles

The average diameter of SNPs, retrieved with TEM images analyses, was (2.6 ± 0.7) nm (300 measurements, Figure S1). TGA analysis of a sample of tA-GNPs under air atmosphere is shown in Figure S2. The formula, calculated based on TEM diameter and TGA analysis, is $Au_{557}(SR)_{163}$. NMR analysis (Figure S3) was used to verify the formation of the thiol monolayer, as proved by the broadening of all signals. The extinction spectra shown in Figure 1 (main text) confirmed the presence of a weak plasmon due to the nucleation and growth of the nanoparticles. The SNPs concentration has been converted into TMAO-SH units grafted on their surface by multiplying by 241.

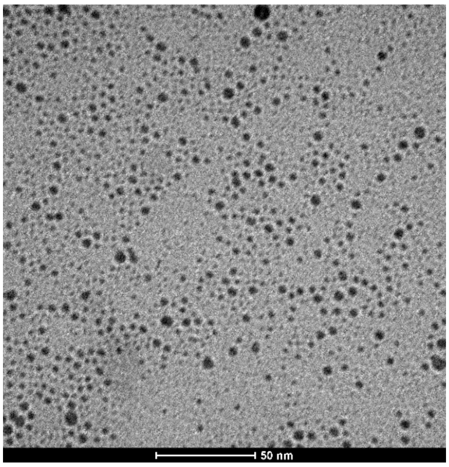

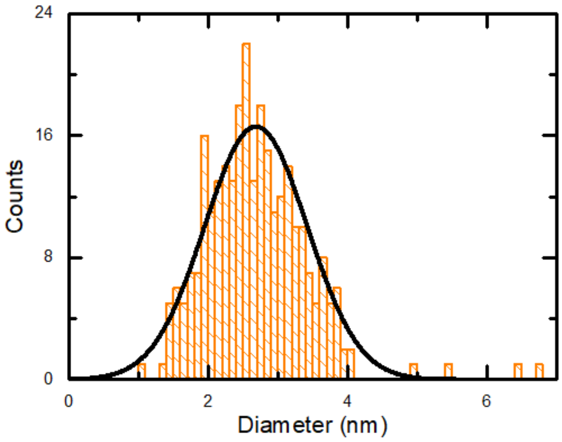

Figure S1. TEM image and analysis of SNPs. The TEM image is reported on the left and size distribution on the right: average diameter is 2.6 nm (σ = 0.7 nm).

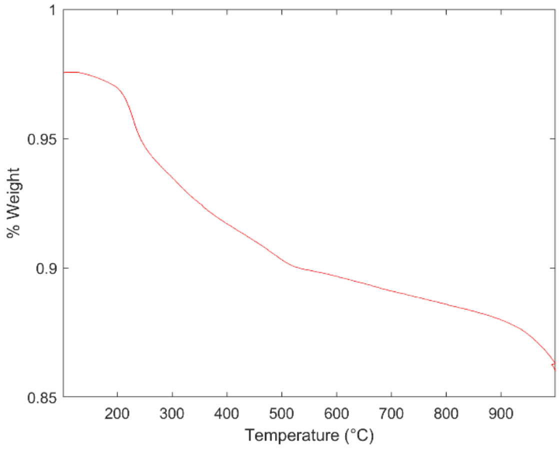

Figure S2. TGA analysis of SNPs. The analysis was carried under air atmosphere.

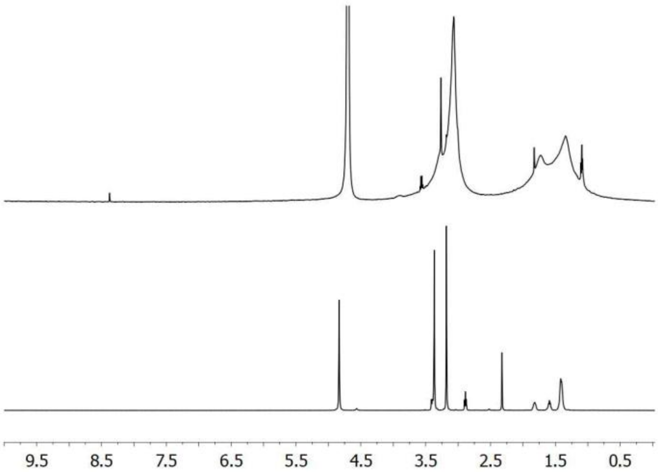

Figure S3. ^{1}H-NMR (500 MHz) spectra of SNPs with 8-trimethylammonium octylthiol in D_2O (upper spectrum) and of 8-trimethylammonium octylthioacetate bromide in MeOD (lower spectrum). The upper spectrum reveals only very broad signals in the same chemical shift regions typical of the coating thiol (1.0–2.0 ppm and 2.5–3.5 ppm). Other signals arise from isotopic impurities of the solvent and traces of other contaminants. This confirms the grafting of the thiol to the nanoparticles (the broadening results from the reduced tumbling rate and mobility of the thiols due to the grafting to the nanoparticles) and their effective purification (no major free organic species are present in the spectrum).

S3. Table of Dynamic Light Scattering Analyses of NPs and Their Hybrids

Table S1. Centroid of the hydrodynamic diameter of BNPs. The relative full width half maximum is reported as error.

Sample	Hydrodynamic Diameter (nm)
BNPs pH = 2.2	12 ± 3
BNPs pH = 11	28 ± 8
BNPs, PAP 3.5, pH = 2.2	32 ± 9
BNPs, PAP 0.35, pH = 2.2	780 ± 180
BNPs, PAP 3.5, pH = 11	1330 ± 120
BNPs, PAP 0.35, pH = 11	200 ± 20

S4. Further Computational Procedures

Two H_2TPPS^{2-} molecules were placed in the same box, initially separated by ~2 nm (two times the cut-off for short-range interactions) and let free to diffuse in solution. Their aggregation occurred within the first 10 ns of simulation and resulted in the formation of a dimer where the electrostatic pairing of SO_3^- groups with the protonated pyrroles dominate (Figure S4a). This structure closely resembles the one commonly assigned to J-aggregates [4]. Quite surprisingly, however, after 150 ns it spontaneously interconverted to a second, less obvious conformation (Figure S4b). The latter shows a different pattern of electrostatic interactions and enhances the hydrophobic effect and steric complementarity: in fact, upon reorganization, the solvent accessible surface area of the dimer decreased from 19.62 nm^2 to 18.11 nm^2. After that, the dimer remained stable until the end of the 1 μs MD. Because of the different interactions at play, we label the dimer structure of Figure S4a as ‘electrostatic’ and the second one in Figure S4b as ‘hydrophobic’.

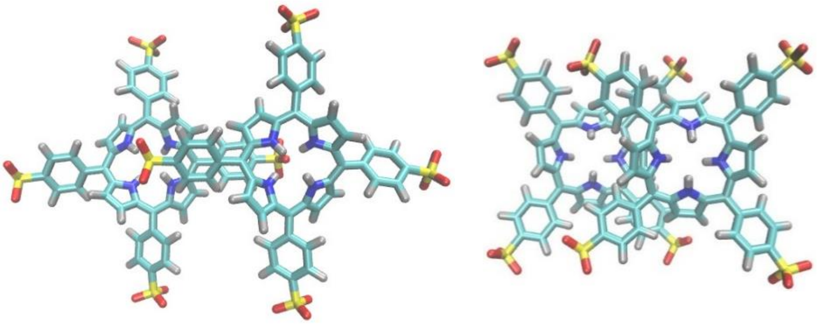

(**a**) (**b**)

Figure S4. Different arrangement of H_2TPPS^{2-} dimer. In particular: in (**a**) the ‘electrostatic’ one is reported, and the ‘hydrophobic’ one in (**b**). As in the following, water and Cl^- ions are not shown for clarity.

The conformational flexibility for the aggregation of H_2TPPS^{2-} molecules was confirmed by eighteen shorter (100–250 ns) MD simulations, where an additional porphyrin was added to either one of the previous dimers: the third molecule could bind either in a pure ‘electrostatic’ or in a ‘hydrophobic’ fashion, regardless of the initial ‘electrostatic’ or ‘hydrophobic’ structure of the starting dimer (Figures S5 and S6). Moreover, while the ‘electrostatic’-to-‘hydrophobic’ rearrangement was occasionally observed, the opposite never happened. Being aware that the actual conformation of large molecular aggregates may be different, since it is the result of a complex growth mechanism [5,6], we decided to proceed with the apparently more stable ‘hydrophobic’ dimer for further investigations.

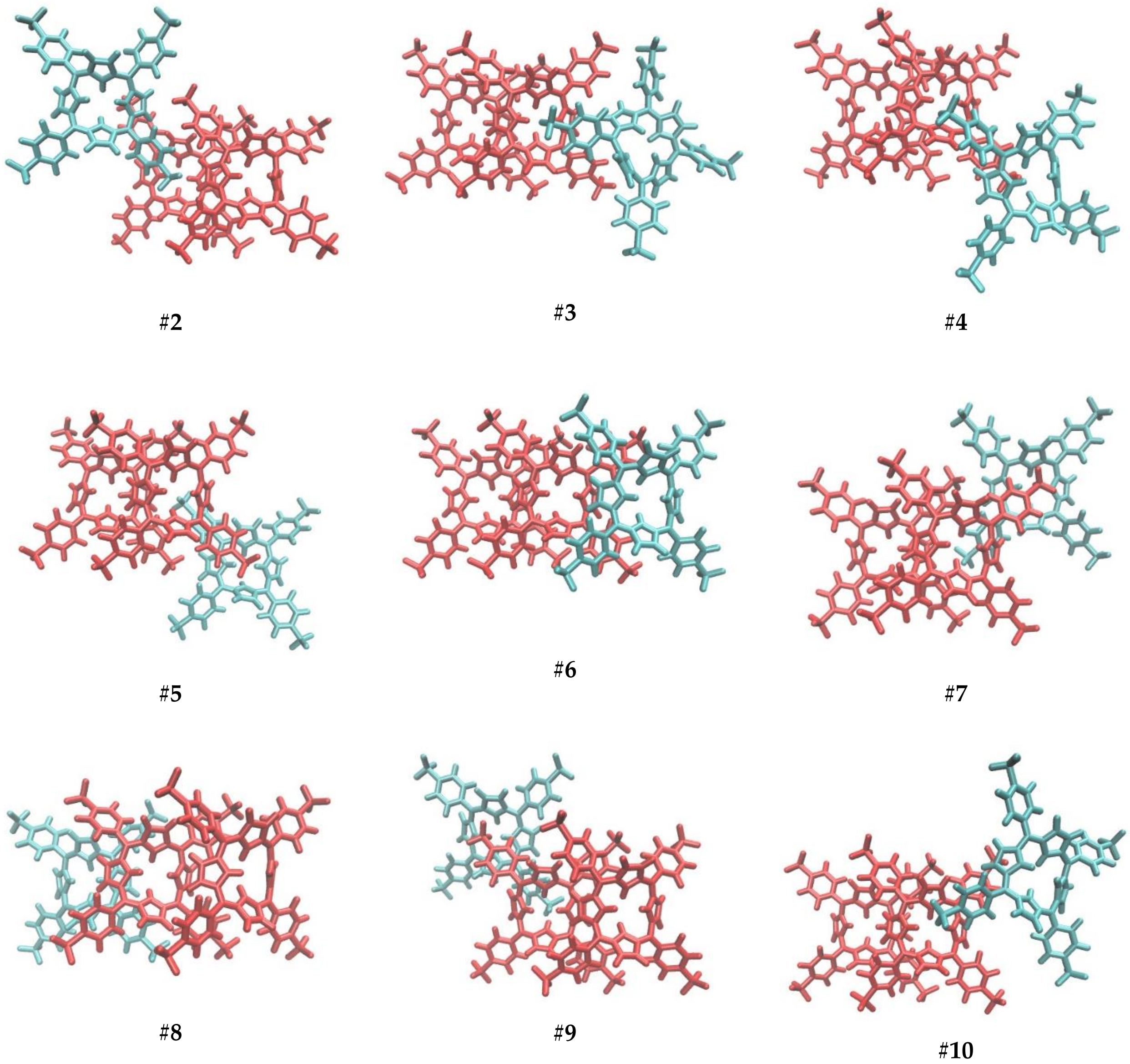

Figure S5. Trimers obtained after the addition of another H_2TPPS^{2-} molecule (cyan) to the 'hydrophobic' dimer (red). The additional porphyrin was initially placed at a distance >1.0 nm (cut-off for van der Waals and short-range electrostatic interactions). At first, MD simulations were run for 100 ns. If the aggregation occurred, they were stopped, otherwise they were extended to 200 ns: after that time, a trimer was obtained for all sample systems. An 'all-hydrophobic' linear trimer was obtained in **6** and **8**; a less compact, bent aggregate, with the third porphyrin bound in an 'electrostatic' fashion, was rather observed in all the remaining cases. The cubic simulation box has an average 7.00 nm side. As in the following, water and Cl^- ions are not shown for clarity.

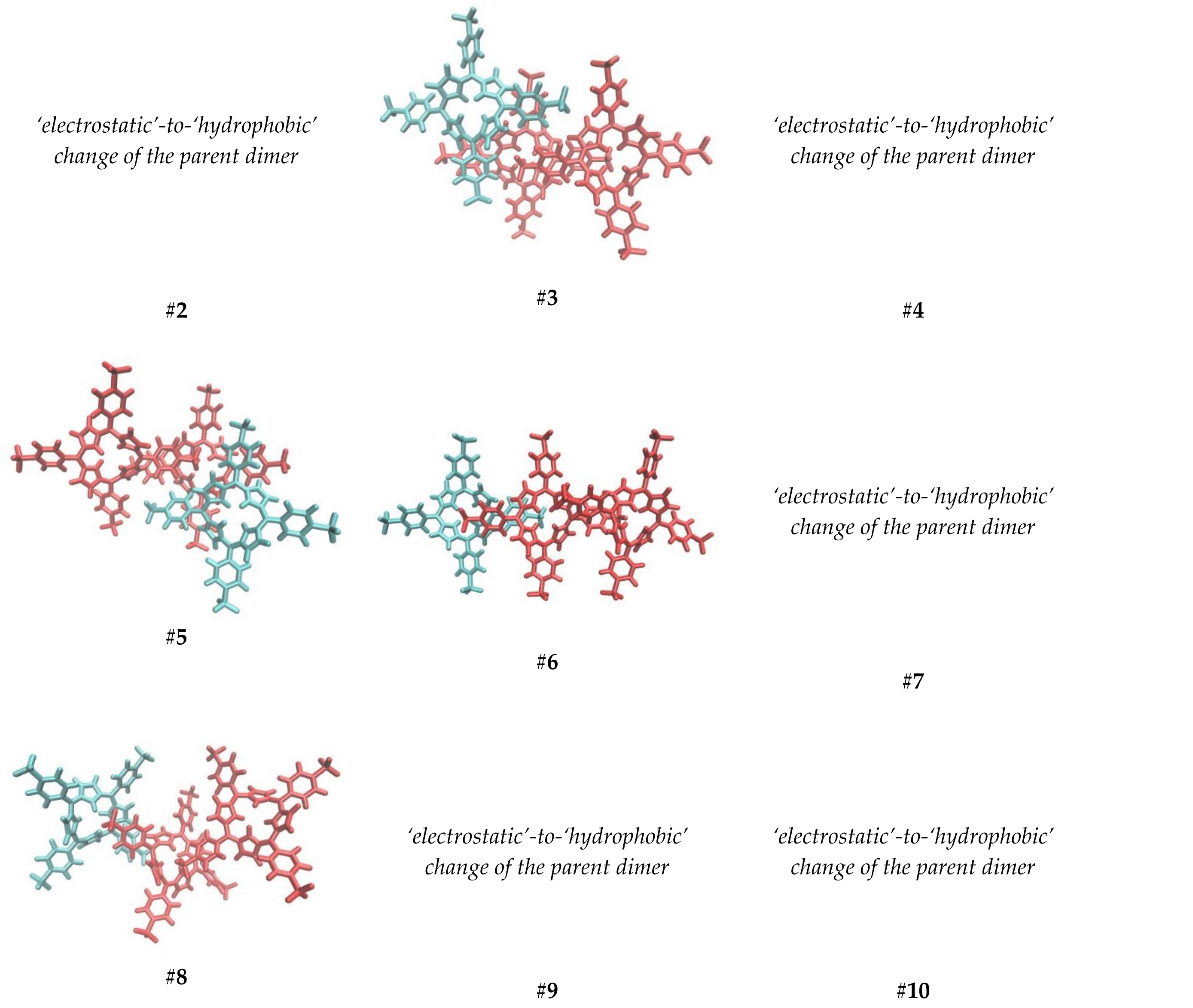

Figure S6. Trimers obtained after the addition of another H_2TPPS^{2-} molecule (cyan) to the 'electrostatic' dimer (red). The additional porphyrin was initially placed at a distance >1.0 nm (cut-off for van der Waals and short-range electrostatic interactions). At first, MD simulations were run for 100 ns. If either aggregation or a change in the starting dimer occurred, they were stopped, otherwise they were progressively extended until a trimer was obtained. In **2**, **4**, **7** and **9** a 'electrostatic'-to-'hydrophobic' interconversion of the parent 'electrostatic' dimer occurred prior to aggregation of the third porphyrin; in **10** an internal rearrangement of the initially formed trimer was observed. While the additional porphyrin binds 'hydrophobically' in **3** and **5**, electrostatic interactions prevail in **6** and **8**. Cubic simulation box has an average 8.03 nm side.

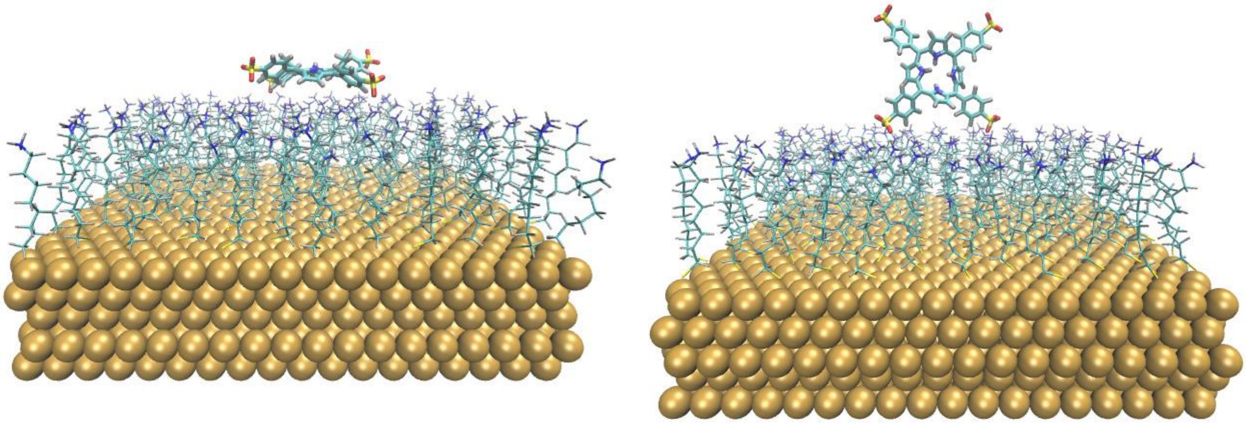

(**a**) (**b**)

Figure S7. Starting configurations (after energy minimization) of a H_2TPPS^{2-} molecule with a single functionalized Au(111) surface. In these MD simulations, the porphyrin was oriented either 'parallel', as in (**a**), or almost perpendicular ('standing'), as in (**b**), with respect to the underlying surface. The Au(111) slab was 5.08 nm × 5.27 nm and is functionalized with 120 ligands. The thickness of the simulated system (including a bare Au(111) surface on top of the solution layer, to avoid a solution-vacuum interface) was fixed to 13.33 nm. The simulation starting from the 'parallel' H_2TPPS^{2-} reached 1 μs, while that for the initially 'standing' molecule was stopped after 600 ns, because the porphyrin got stuck on the bare surface at the top of the simulation box. In fact, during both dynamics, the porphyrin repeatedly moved back and forth to the surface, recreating the binding interactions with the SAM multiple times. This evidence comes out in favour of a satisfactory sampling.

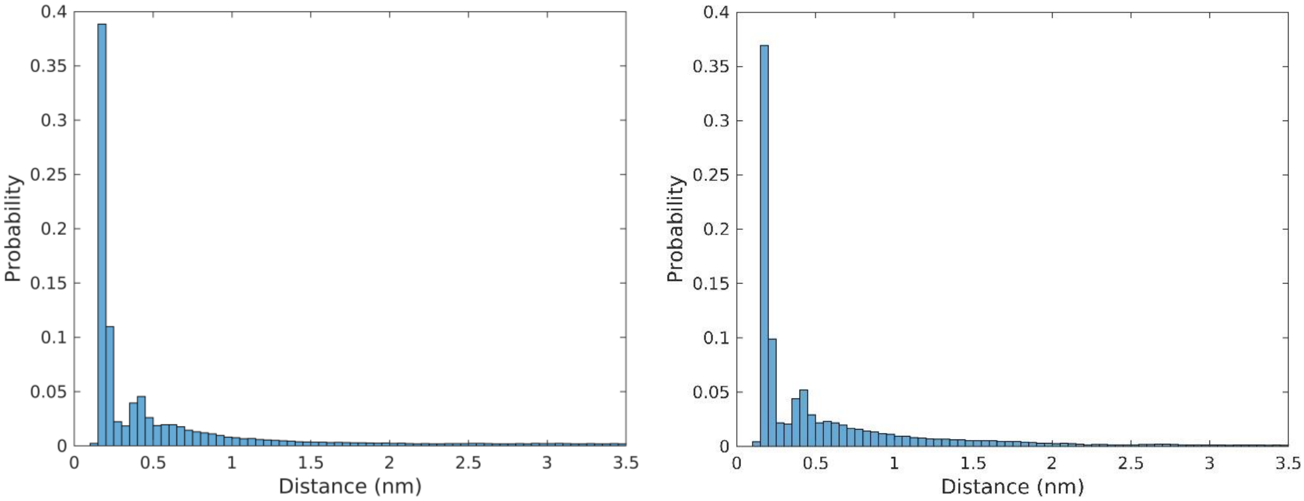

(**a**) (**b**)

Figure S8. H_2TPPS^{2-}-ligand distance of one porphyrin with a single functionalized Au(111) surface. In these histograms referred to the MD simulations, (**a**) shows the result for the 1 μs trajectory starting from the 'parallel' porphyrin; (**b**) shows the result for the 600 ns trajectory obtained from the 'standing' molecule.

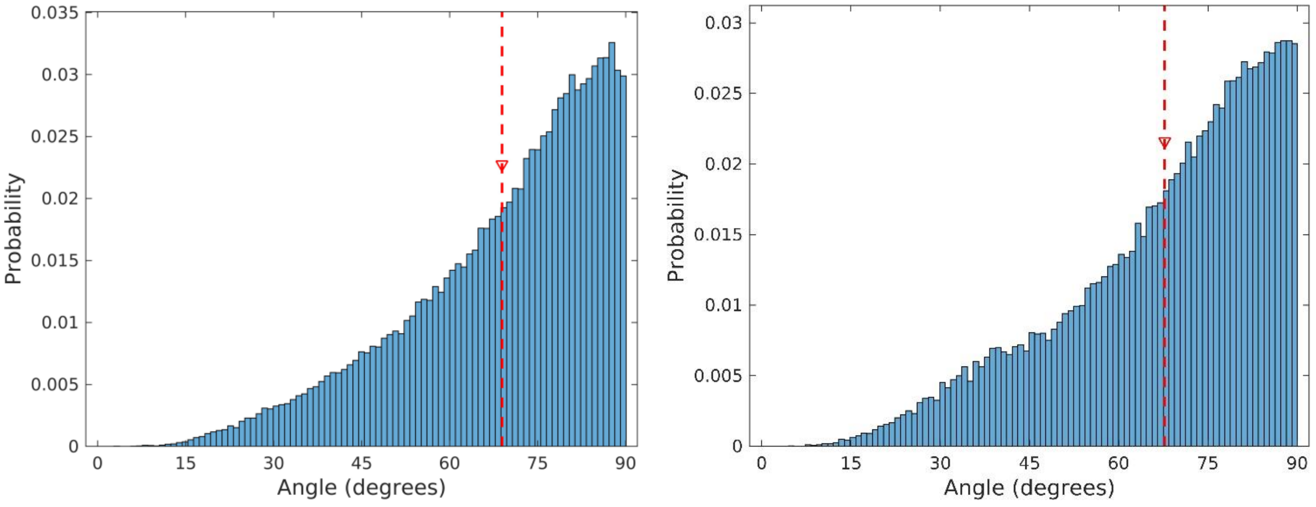

(**a**) (**b**)

Figure S9. H_2TPPS^{2-}-ligand angle of one porphyrin with a single functionalized Au(111) surface. In these histograms referred to the MD simulations, the angle is defined as the H_2TPPS^{2-} plane and the z-axis (i.e., to the normal vector of the underlying surface). The red dashed line indicates the average value. (**a**) shows the result for the 1 μs trajectory starting from the 'parallel' porphyrin; (**b**) shows the result for the 600 ns trajectory obtained from the 'standing' molecule.

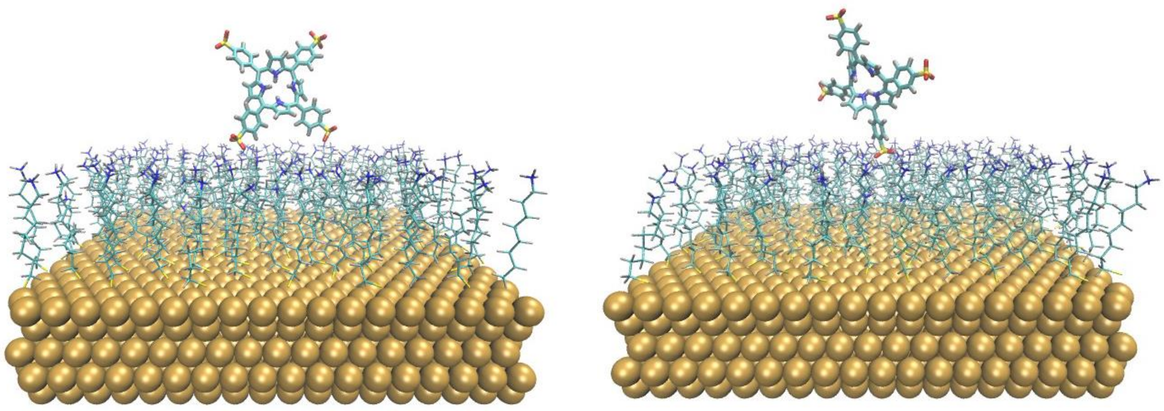

(**a**) (**b**)

Figure S10. Two representative clusters obtained starting for the initially 'standing' porphyrin molecule, and with a bidentate coordination initially enforced. These two representative clusters were obtained from the shorter (600 ns) MD simulations. They account for 33.3% (**a**) and 10.3% (**b**) of the analysed frames.

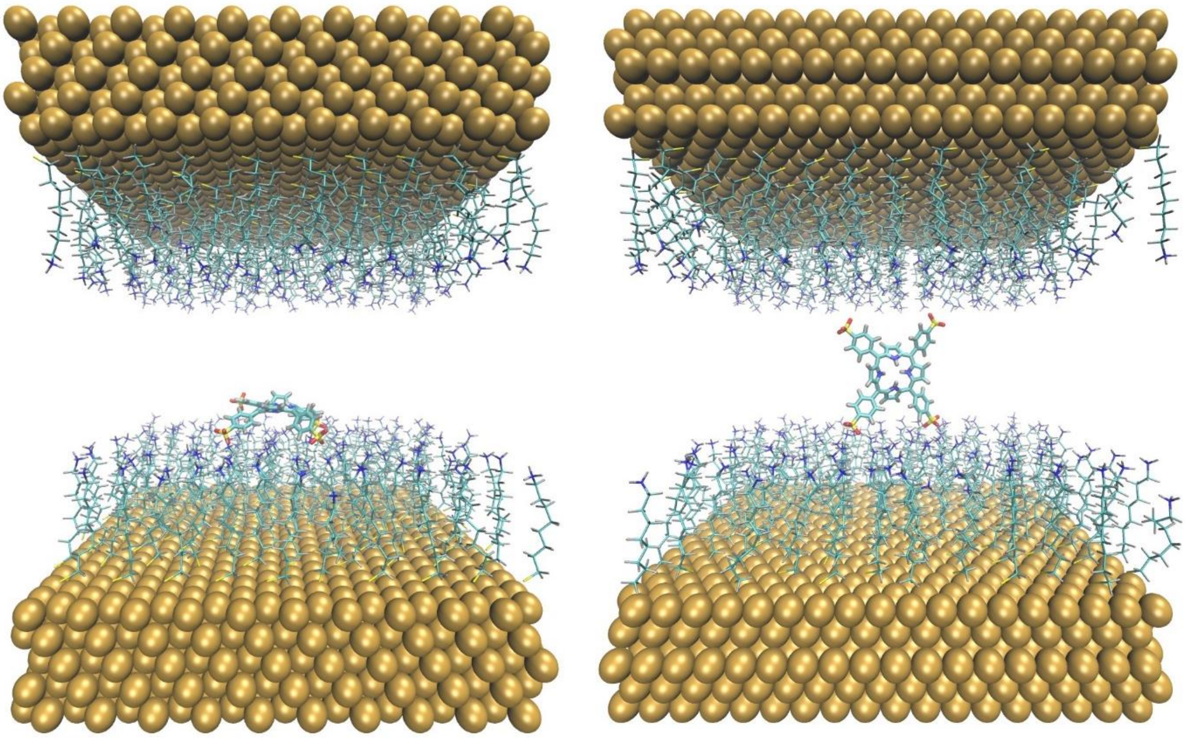

(**a**) (**b**)

Figure S11. Starting configurations (after energy minimization) for the two 1 μs MD simulations of a H_2TPPS^{2-} molecule between two capped Au(111) surfaces. The porphyrin was oriented either in a 'parallel' (**a**), or in an almost perpendicular ('standing') fashion (**b**), with respect to the underlying surface. Each Au(111) slab was 5.08 nm × 5.27 nm and is functionalized with 120 ligands. The thickness of the simulated system was fixed to 6.69 nm. The starting configurations for the shorter (500 ns) simulations with a thinner solution layer were obtained by lowering the upper surface by 0.20 nm.

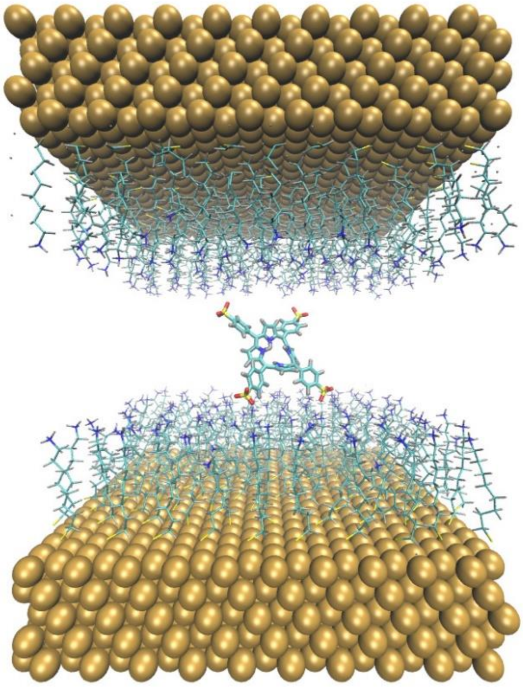

Figure S12. A representative cluster, starting the MD simulation from a 'parallel' H_2TPPS^{2-} molecule between two functionalized Au(111) surfaces, in the thicker box. This representative cluster accounts for 25.3% of the analysed frames in the 1 μs MD simulation. While two SO_3^- ••• NH_3^+ contacts are fully exploited, the other two, involving the upper surface, are slightly above the threshold value of 0.30 nm (0.35–0.40).

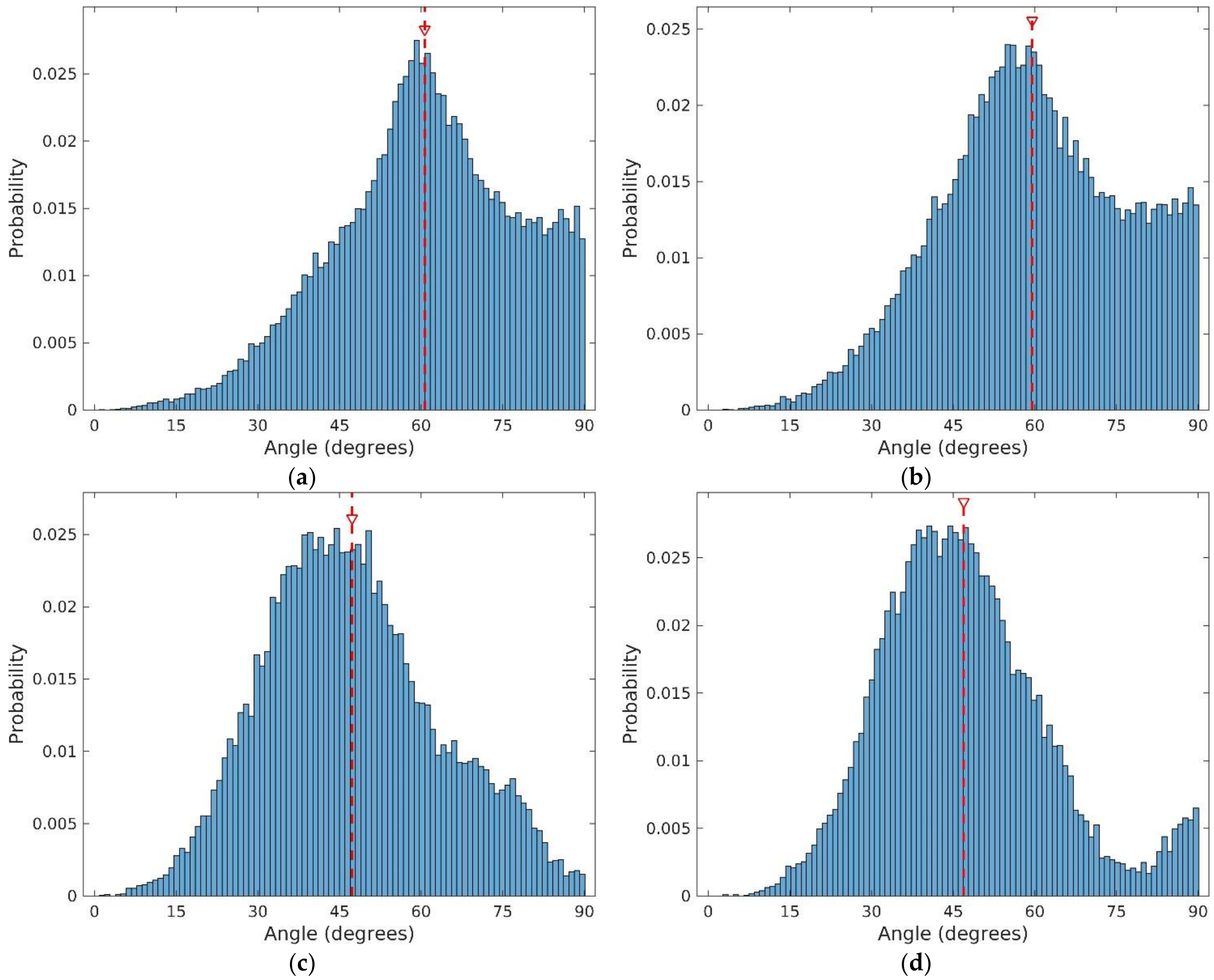

Figure S13. H_2TPPS^{2-}-ligand angle of one porphyrin between two functionalized Au(111) surface. In these histograms referred to the MD simulations, the angle is defined as the H_2TPPS^{2-} plane and the *z*-axis (i.e., to the normal vector of the underlying surface). The red dashed line indicates the average value. A 0° angle corresponds to the dye molecule laying down parallel to the surface, while a right angle indicates a perpendicular orientation. (**a**) and (**c**) show the results for the trajectories starting from the 'parallel' porphyrin (1 μs in the thicker box and 500 ns in the 0.2 nm thinner box, respectively); (**b**) and (**d**) show the results for the trajectories obtained from the 'standing' molecule (1 μs in the thicker box and 500 ns in the 0.2 nm thinner box, respectively).

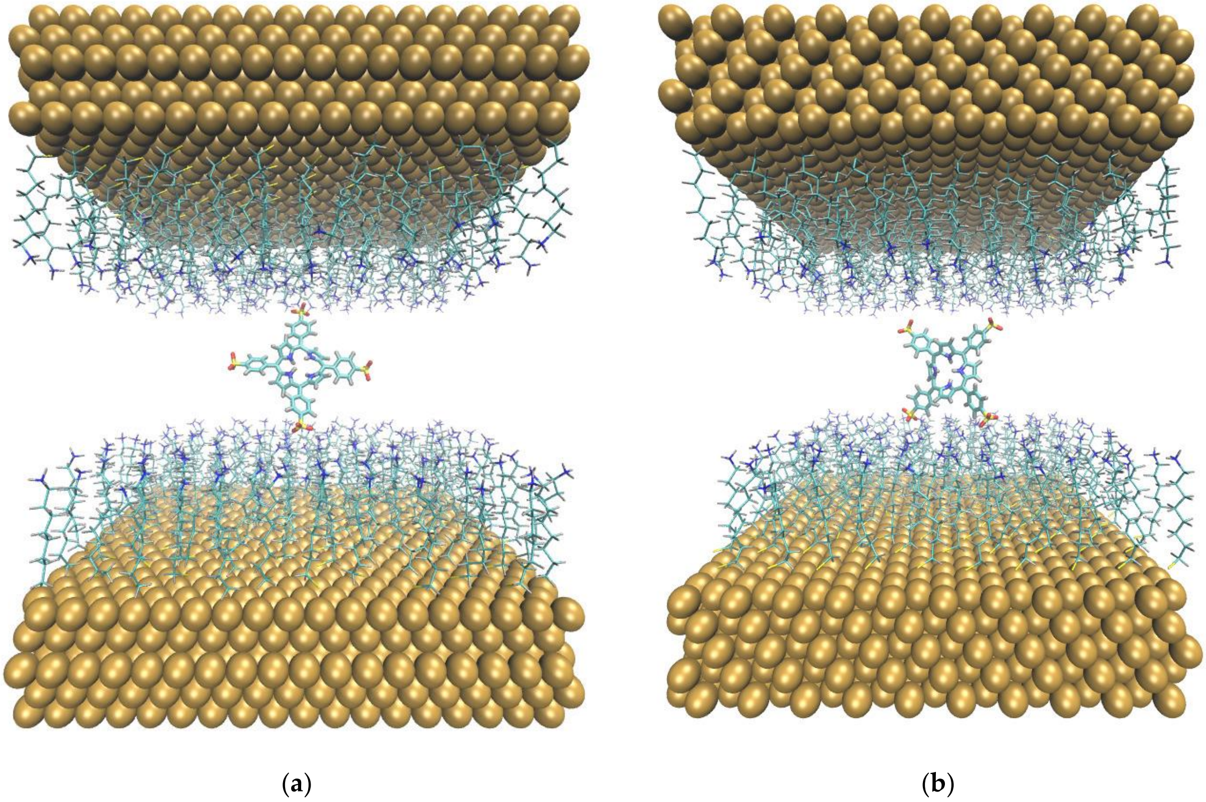

Figure S14. Two representative clusters of H_2TPPS^{2-} molecule between two functionalized Au(111) surfaces. These clusters are retrieved from the 1 μs simulation starting from a 'standing' H_2TPPS^{2-} configuration in the thicker box. (**a**) and (**b**) account for 25.8% and 7.3% of the analysed frames, respectively. In (**b**), while two SO_3^- ••• NH_3^+ contacts are fully exploited, the other two, involving the upper surface, are slightly above the threshold value of 0.30 nm (0.30–0.35 nm).

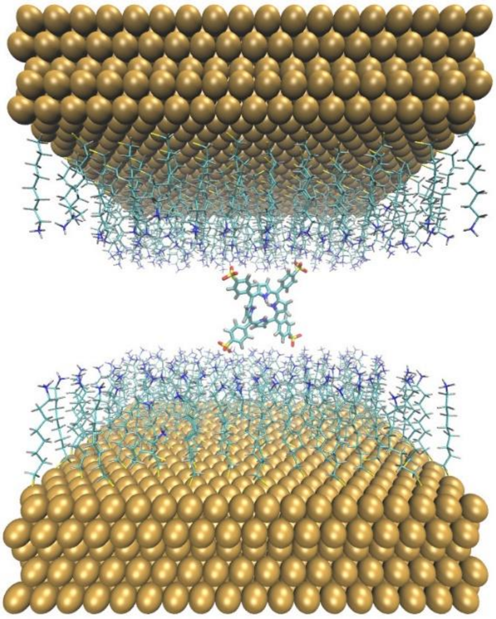

Figure S15. A representative cluster of H_2TPPS^{2-} molecule between two functionalized Au(111) surfaces, in a 0.2 nm thinner box. It accounts for 24.9% of the analysed frames in the 500 ns MD simulation starting from a 'standing' H_2TPPS^{2-} molecule. All the four possible SO_3^- ••• NH_3^+ contacts are exploited. Each Au(111) slab was 5.08 nm × 5.27 nm and is functionalized with 120 ligands. The thickness of the surface-solution-surface system was fixed to 6.49 nm.

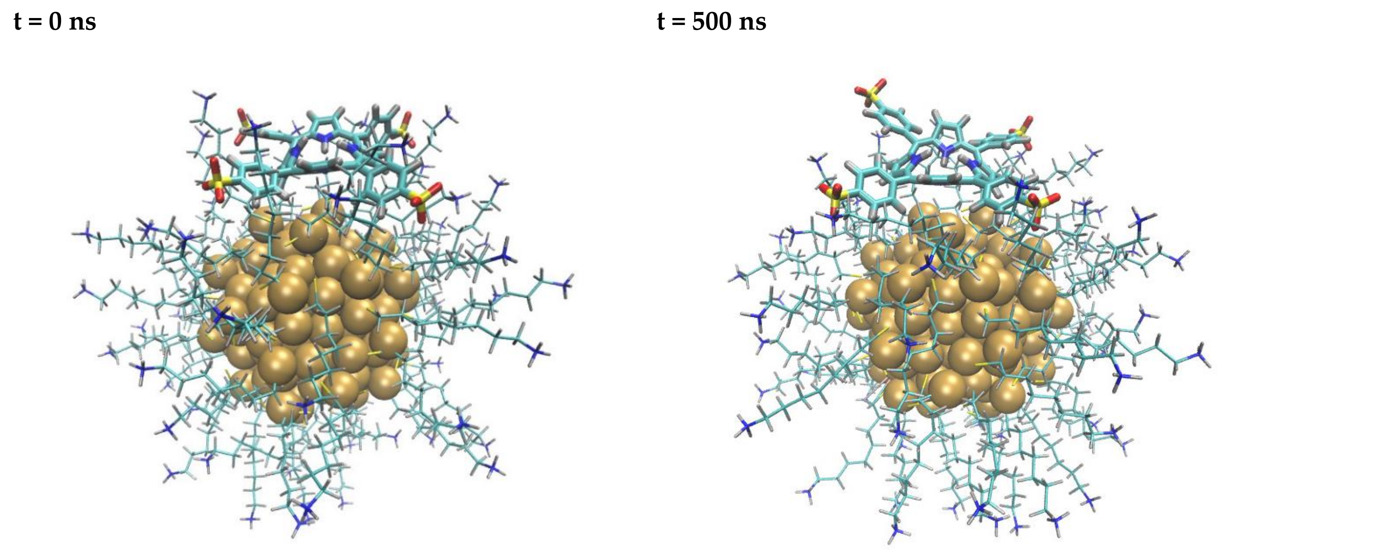

Figure S16. Initial and final configurations for the additional 500 ns MD simulations of an H_2TPPS^{2-} molecule with the Au_{144}(S-AO)$_{60}$ nanocluster. The simulations started after the addition of counter-ions to neutralize the box. The cubic simulation box has a fixed 8.49 nm side.

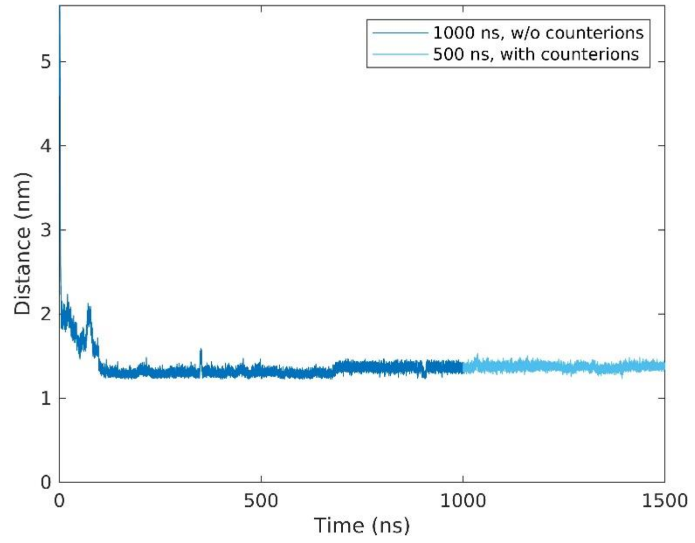

Figure S17. Plot of the c.o.m.–c.o.m. distance between the Au_{144}(–S-AO)$_{60}$ nanocluster and a H_2TPPS^{2-} molecule. The dark blue line refers to the initial 1 μs MD simulation without counterions in the box; the light blue one refers to the additional 500 ns dynamics after neutralization of the box.

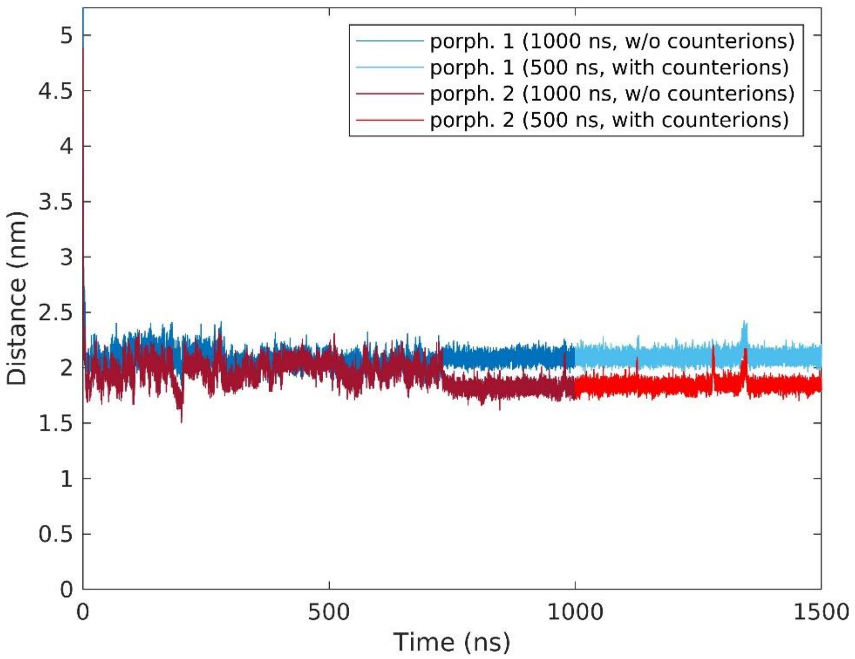

Figure S18. Plot of the c.o.m.–c.o.m. distance between the Au_{144}(–S-AO)$_{60}$ nanocluster and each of the H_2TPPS^{2-} residues in a 'hydrophobic' dimer. The dark red and dark blue lines refer to the initial 1 μs MD simulation without counterions in the box; the light red and light blue lines to the additional 500 ns dynamics after the neutralization of the box.

t = 0 ns **t = 500 ns**

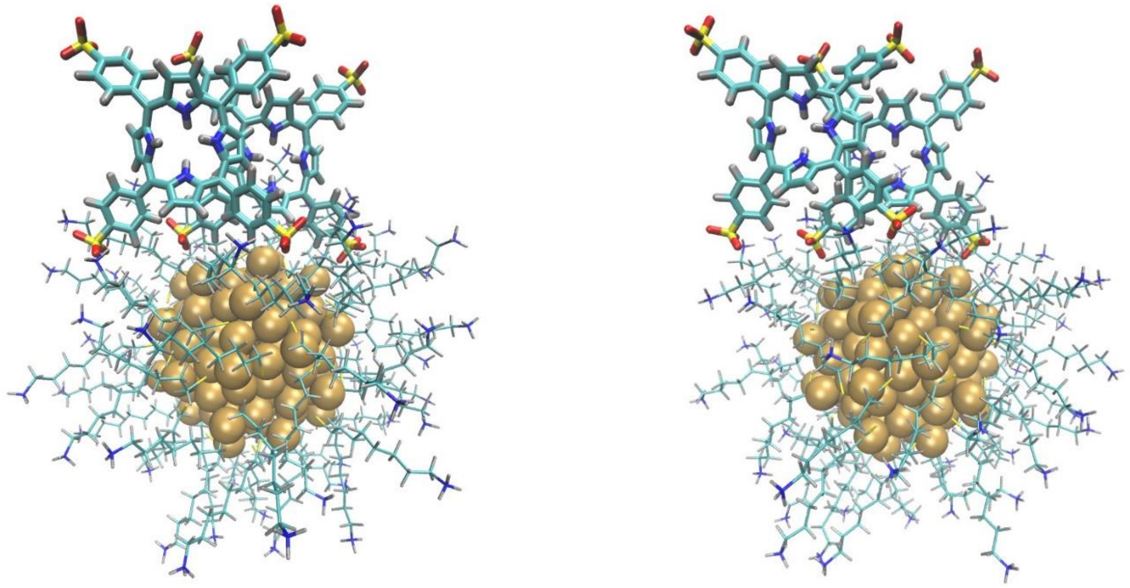

Figure S19. Initial and final configurations for the additional 500 ns MD simulations of the 'hydrophobic' dimer with the Au_{144}(–S-AO)$_{60}$ nanocluster. The simulations stated after the addition of counterions to neutralize the box. The cubic simulation box has a fixed 9.16 nm side.

S5. Calculation of the H_2TPPS^{2-} Configuration on BNPs Surface

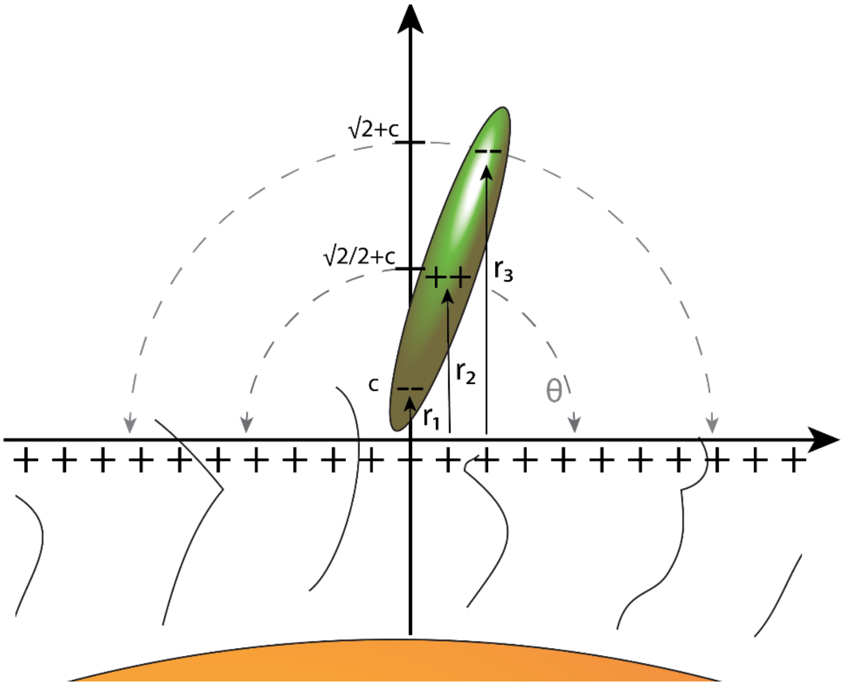

Figure S20. 2D representation of H_2TPPS^{2-} on a BNP surface. The y and x axes indicate the distance expressed in nm to the point of contact between the two nanoobjects.

H_2TPPS^{2-} geometry can be approximated as a square with a side of $\sqrt{2}$ nm that bears four negative charges at the corner and two positive charges in its centre [7]. Since SNPs and BNPs diameter are at least twice H_2TPPS^{2-} length, we approximated their interactions with H_2TPPS^{2-} as the interaction of a plane with a homogeneous charge density. In Figure S20 we represented it. On the y axis we reported the distance to the surface. Considering an H_2TPPS^{2-} grafted to the BNP surface with two sulfonate groups, simply trigonometrical considerations allowed us to calculate the distances of the further negative charges, the positive charges and the closer negative charges, respectively, as:

$$r_3 = \sqrt{2}\,\mathrm{sen}(\theta) + c \tag{S1}$$

$$r_2 = \frac{\sqrt{2}}{2}\mathrm{sen}(\theta) + c \tag{S2}$$

$$r_1 = c \tag{S3}$$

with c the distance between the trimethyl ammonium cationic layer and the sulfonate groups, which is the sum of their van der Waals radii. Being the electric potential of a plane with a homogeneous charge density V proportional to the opposite of the distance from it and the general formula for the electric potential energy

$$U_e = \frac{1}{2}qV; \tag{S4}$$

with q the value of the electric charge, we calculated U_e as:

$$U_e \propto \frac{1}{2}\left(-2qr_1 + 2qr_2\right). \tag{S5}$$

The first term within the brackets is the contribution of the protonated pyrrolic groups, while the second term is the contribution of the sulfonate groups. By substituting (S1) and (S2) in (S5), it follows that:

$$U_e \propto sen(\theta). \quad \text{(S6)}$$

From these considerations it follows that the minimum of H_2TPPS^{2-} potential energy is reached when the porphyrin is parallel to the NPs surface, while its maximum is with perpendicular orientation.

References

1. Bonomi, R.; Cazzolaro, A.; Prins, L.J. Assessment of the Morphology of Mixed SAMs on Au Nanoparticles Using a Fluorescent Probe. *Chem. Commun.* **2011**, *47*, 445–447, https://doi.org/10.1039/c0cc02260h.
2. Peruffo, N.; Gil, G.; Corni, S.; Mancin, F.; Collini, E. Selective Switching of Multiple Plexcitons in Colloidal Materials: Directing the Energy Flow at the Nanoscale. *Nanoscale* **2021**, *13*, 6005–6015, https://doi.org/10.1039/d1nr00775k.
3. Manea, F.; Bindoli, C.; Polizzi, S.; Lay, L.; Scrimin, P. Expeditious Synthesis of Water-Soluble, Monolayer-Protected Gold Nanoparticles of Controlled Size and Monolayer Composition. *Langmuir* **2008**, *24*, 4120–4124, https://doi.org/10.1021/la703558y.
4. Akins, D.L.; Zhu, H.-R.; Guo, C. Absorption and Raman Scattering by Aggregated meso-Tetrakis(p-Sulfonatophenyl)Porphine. *J. Phys. Chem.* **1994**, *98*, 3612–3618, https://doi.org/10.1021/j100065a012.
5. Aggarwal, L.P.F.; Borissevitch, I.E. On the Dynamics of the $TPPS_4$ Aggregation in Aqueous Solutions: Successive Formation of H and J Aggregates. *Spectrochim. Acta A* **2006**, *63*, 227–233, https://doi.org/10.1016/j.saa.2005.05.009.
6. Romeo, A.; Castriciano, M.A.; Occhiuto, I.; Zagami, R.; Pasternack, R.F.; Scolaro, L.M. Kinetic Control of Chirality in Porphyrin J-Aggregates. *J. Am. Chem. Soc.* **2013**, *136*, 40–43, https://doi.org/10.1021/ja410514k.
7. Fleischer, E.B. Structure of Porphyrins and Metalloporphyrins. *Accounts Chem. Res.* **1970**, *3*, 105–112, https://doi.org/10.1021/ar50027a004.
